# Complete Genome Sequence of an Attenuated Duck Enteritis Virus Obtained by *In Vitro* Serial Passage

**DOI:** 10.1128/genomeA.00685-13

**Published:** 2013-09-05

**Authors:** Chenghuai Yang, Junping Li, Qihong Li, Huijiao Li, Yecai Xia, Xin Guo, Kangzhen Yu, Hanchun Yang

**Affiliations:** Key Laboratory of Animal Epidemiology and Zoonosis of the Ministry of Agriculture, College of Veterinary Medicine and State Key Laboratory of Agrobiotechnology, China Agricultural University, Beijing, People’s Republic of Chinaa; China Institute of Veterinary Drug Control, Beijing, People’s Republic of Chinab; Ministry of Agriculture, Beijing, People’s Republic of Chinac

## Abstract

Here, we present the complete genome sequence of an attenuated duck enteritis virus (DEV) obtained by serial chicken embryo passage. Compared with a virulent DEV, there is a serial deletion in unique long open reading frame 11 (LORF11) and unique long region 2 (UL2). This study will aid in further exploration of the molecular pathogenesis of DEV.

## GENOME ANNOUNCEMENT

Duck viral enteritis (DVE), also known as duck plague, is an acute, contagious, and lethal disease of ducks, geese, and swans. The disease can cause high mortality and decreased egg production in domestic and wild waterfowl, resulting in significant economic losses (1). The causative agent, duck enteritis virus (DEV), is taxonomically classified as a new species, *Anatid herpesvirus 1*, in the genus *Mardivirus*, subfamily *Alphaherpesvirinae*, and family *Herpesviridae* (2).

DEV strain K at passage 63 was first isolated in southern China’s Guangdong province in 1957 (3) and had been serially passaged >60 times in chicken embryo to attenuate its virulence (unpublished data). The 63rd-passage virus, obtained from the China Center for Veterinary Culture Collection (CVCC) (Beijing, China), is lethal to chicken embryos, and the 50% embryo lethal dose was 10^-7.6^/0.2 ml; however, it is avirulent for ducks 2 months old and older.

DEV strain K DNA was extracted from infected chicken embryo fibroblasts as described previously (4). Sequencing of 5.0 µg DNA was carried out commercially on a Genome Sequencer 20 (GS20) system (454 Life Sciences Corporation). DNA sequences were assembled using the SeqMan program (v7.0; DNAStar) and edited manually. The complete genome sequence of the 63rd-passage strain is 158,089 nucleotides (nt). A total of 78 open reading frames (ORFs) were predicted to encode a potential functional protein. There is very high identity between the 63rd-passage strain and DEV strain VAC (5), with a difference of only 44 single-nucleotide polymorphisms. Compared with the virulent DEV strains CHv (6, 7) and 2085 (8), the 63rd-passage strain had a 3,513-bp deletion and a 2,497-bp deletion, respectively, after nucleotide 2715, causing a frameshift in long ORF 11 (LORF11). Additionally, there was a 528-bp deletion that caused the deletion of 176 amino acids in the frame of unique long region 2 (UL2). The reason for the differences in genomic sequences, the pathogenic potency, and the geographic origin are still unknown.

In all, our work will be helpful for further exploring the genetic diversity during *in vitro* serial passage and the molecular pathogenesis of DEV.

### Nucleotide sequence accession number.

The complete genome sequence for the 63rd passage of DEV strain K in chicken embryo is available in GenBank under the accession no. KF487736.
